# A Semi-Active Control Technique through MR Fluid Dampers for Seismic Protection of Single-Story RC Precast Buildings

**DOI:** 10.3390/ma15030759

**Published:** 2022-01-19

**Authors:** Nicola Caterino, Mariacristina Spizzuoco, Valeria Piccolo, Gennaro Magliulo

**Affiliations:** 1 Department of Engineering, University of Naples Parthenope, 80100 Naples, Italy; valeria.piccolo001@studenti.uniparthenope.it; 2 Institute of Technologies for Construction, National Research Council (CNR), 20098 Novate Milanese, Italy; 3 Department of Structures for Engineering and Architecture, University of Naples Federico II, 80100 Naples, Italy; spizzuoc@unina.it (M.S.); gennaro.magliulo@unina.it (G.M.)

**Keywords:** semi-active control, magnetorheological dampers, precast RC buildings, seismic risk

## Abstract

The work proposes an innovative solution for the reduction of seismic effects on precast reinforced concrete (RC) structures. It is a semi-active control system based on the use of magnetorheological dampers. The special base restraint is remotely and automatically controlled according to a control algorithm, which modifies the dissipative capability of the structure as a function of an instantaneous dynamic response. The aim is that of reducing the base bending moment demand without a significant increase in the top displacement response. A procedure for the optimal calibration of the parameters involved in the control logic is also proposed. Non-linear modelling of a case-study structure has been performed in the OpenSees environment, also involving the specific detailing of a novel variable base restraint. Non-linear time history analyses against natural earthquakes allowed testing of the optimization procedure for the control algorithm parameters, finally the capability of the proposed technology to mitigate seismic risk of new or existing one-story precast RC structures is highlighted.

## 1. Introduction

The recent seismic events that occurred in Italy, e.g., the 2012 Emilia earthquake [1], caused extensive damage to industrial single-story reinforced concrete (RC) precast buildings. The most relevant structural damage shown by this type of building during the post-event surveys was the failure of beam-to-column and beam-to-roof tiles connections [2]; yielding or large cracking at the column bases was detected too. Most of the beam-to-column and beam-to-roof tiles connections of the existing Italian single-story RC precast buildings are based on friction forces because they were built when seismic regulations were not in force in Italy or when friction connections were allowed in seismic areas too. Sometimes, the pinned connections, generally made of one or more dowels cast in the column and inserted in holes pre-formed in the beam and fixed by a final grout cast, showed damage too, mainly due to the lack of adequate dowel cover, which isn’t ruled by any Italian code, neither in the past nor currently.

In general, the seismic behavior of these types of buildings can be improved by rethinking the structural system (e.g., with the addition of shear walls, conventional [3] or dissipating rocking walls [4]; by installing exoskeletons in steel [5] or aluminum [6]) as well as with low impact interventions. In recent years, the most common intervention for increasing the seismic safety of existing single-story RC precast buildings, has been the increase of the connections’ strength by mechanical devices [7,8,9,10,11]. This intervention was even imposed by the Italian government in the area struck by the Emilia earthquake immediately [12].

However, the increment of the seismic capacity of the beam-to-column (and beam-to-roof tiles) connections often does not significantly increase the seismic capacity of the whole existing single-story RC precast building. This is because such intervention generally causes an increase in demand to columns under seismic actions, leading to the development of a plastic hinge at the base or a rigid rotation when foundation issues arise. In the best case, the column’s strength is sufficient to withstand the seismic forces, however earthquake loads cause large lateral displacements, with the consequence of the disrupting the use and/or the failure of nonstructural elements, e.g., of the cladding panels.

An innovative solution is presented herein, based on seismic load filtering at the base of the columns. It consists of the weakening, rather than strengthening, of the column-to-foundation connection. A deformable link at the base of the columns allows rotation (rocking) around the horizontal axis. This can be utilized in the case of an existing building where a flexural disconnection is realized between the column and its foundation, however the proposed solution is also suitable for new buildings. The base rocking is contrasted through a spring that is locally installed and exploited for energy dissipation (i.e., reduction of seismic demand) by means of a semi-active (SA) control system, based on the use of magnetorheological (MR) dampers. The above strategy is applied to a single-story RC precast building as case-study.

In recent years, many studies have explored the idea of using a rocking mechanism between precast elements to dissipate energy [11,12,13,14,15,16,17,18,19,20,21], however, to the authors’ knowledge, none of them include a smart control system. The main advantage of a SA control with variable dampers is the ability to modify the mechanical properties of the devices according to a given control logic that aims at reducing the overall structural seismic response. In particular, the goal of the SA system proposed herein, is that of limiting the bending moment at the base of the columns and displacement at the top of the building. A similar strategy has been already applied successfully to mitigate wind induced structural demand in wind turbines [22].

In the proposed study, the MR device is driven through a control logic originally proposed in [23]. Nonlinear time-history analyses of the case study are performed to calibrate the control algorithm and to assess the effectiveness of the technique.

## 2. The Proposed Control Strategy

**Figure 1:**
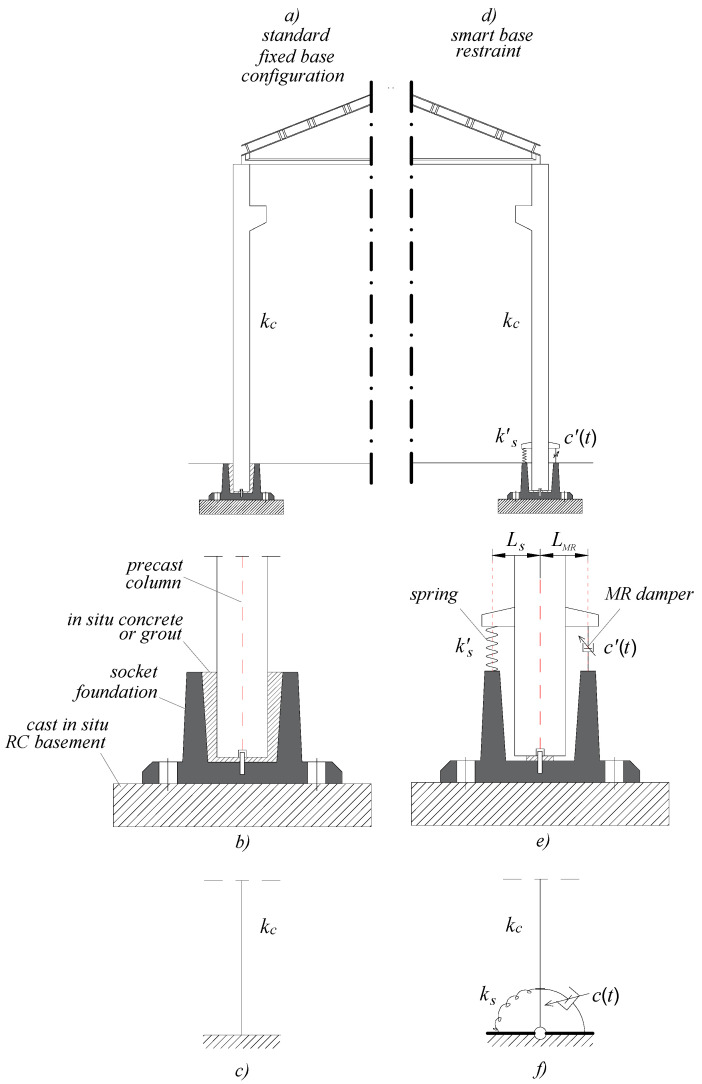
Schematic representation of the base restraint: standard (overall view (**a**), focus onto the base connection (**b**), structural model (**c**)) and smart (overall view (**d**), focus onto the base connection (**e**), structural model (**f**)) configurations.

The idea is schematically represented in Figure 1. On the left, the structural configuration of a standard one-storey precast RC structure is shown (Figure 1a–c). The column is connected to the cast-in-situ RC basement through a precast socket foundation with a final completion of casting of concrete or cementitious mortar (Figure 1b). In this case, the idea is that of making the base restraint stiff and resistant enough as a fixed support (Figure 1c). On the right side of Figure 1, the smart base restraint is sketched. The column is hinged at the base, allowing rocking movement around the horizontal axes passing through the base steel dowel. This movement is partially contrasted by a vertical spring (Figure 1e; stiffness *k’_s_*), stiff enough to limit base rotation, therefore top displacement demand, to within acceptable limits. Such elastic base rotation puts the MR damper in action. This exploits the movement dissipating a large amount of energy, utilizing the control logic that optimally calibrates its damping capability *c’*(*t*) for each instant of time t. Figure 1f shows how this special semi-active base restraint can be modelled, i.e., with a rotational spring in parallel with a rotational MR damper, with *k_s_* = *k’_s_*·*L_s_* and *c*(*t*) = *c’*(*t*) *L_MR_*. It is worth highlighting that the technological and implementation aspects will be addressed in greater detail in subsequent studies. The mechanical connection of RC columns to MR dampers and springs is realized by means of RC corbels (brackets), that are short-haunched cantilevers, similar to those commonly used to support beams in pre-cast structural systems.

As will be described in greater detail below, the control logic is designed to make the base restraint behave in two different ways, rigid and dissipative, switching instant by instant with the aim of reducing, as much as possible, the structural response:

- In the rigid configuration (“fixed base”, (FB)), the MR devices are powered with the maximum current (briefly, state “ON” in the following) and therefore exhibit a very rigid behavior; this, in essence, limits the base rotation;
- In the dissipative configuration (“semi-active”, (SA)), the devices are fed with a lower intensity (briefly, state “OFF” in the following) and then basically behave like non-linear viscous dampers; in this case, the base rotates, the springs react elastically, and the dampers dissipate energy, thus reducing the structural response.

**Figure 2:**
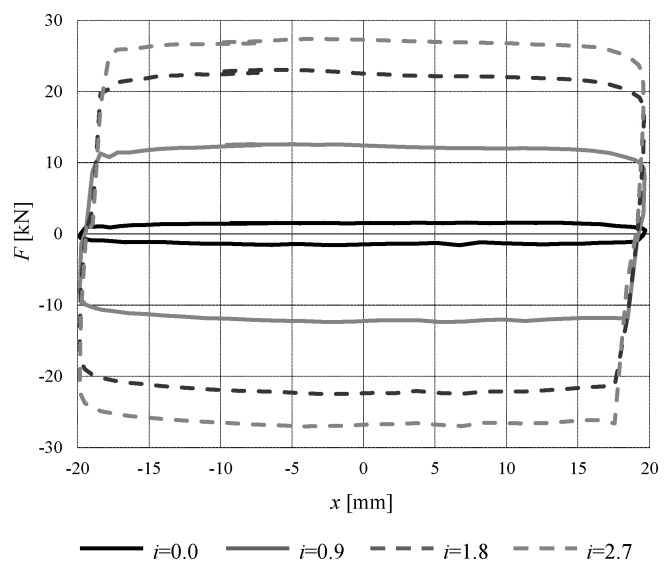
Experimental force-displacement loops (1.5 Hz, ±20 mm) for a prototype MR damper at different levels of current (Ampere) [24].

To give an example of the variability of the mechanical behavior of an MR device, Figure 2 shows the cyclic behavior of a device tested by the authors [24]. It can be observed that, with the same displacement imposed (cyclic at 1.5 Hz, ±20 mm), the device reacts very differently depending on the current that feeds it, and therefore on the intensity of the magnetic field inside it. In this specific case, the maximum level of force varies from 2 kN (intensity of current 0 A) to 27 kN (2.7 A). The above introduced state “ON” and state “OFF” of the MR device must be intended as corresponding to a high value and a low value of the feeding current, respectively.

**Figure 3:**
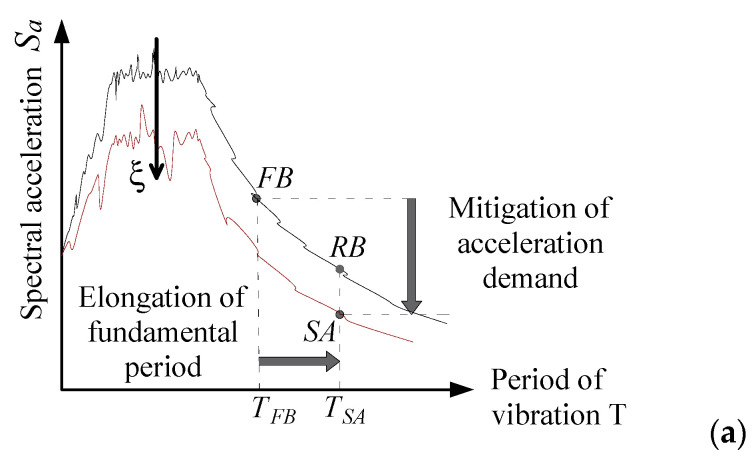

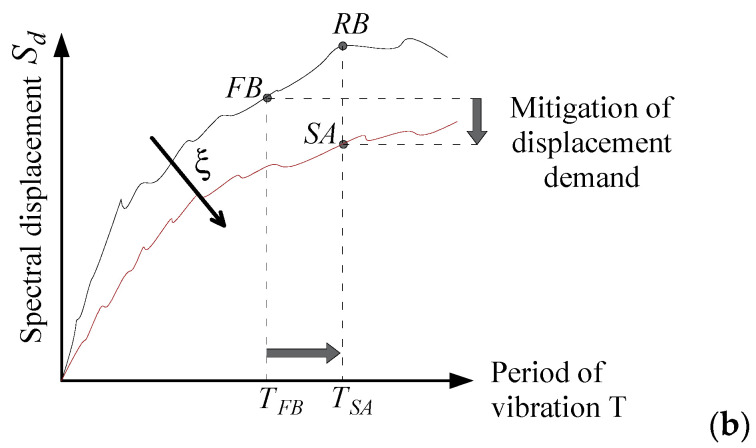
Reduction of seismic demand from FB to SA configuration, ideally passing through an intermediate state RB without additional damping: acceleration (**a**) and displacement (**b**) spectra.

When the restraints at the base of the structure change from a FB configuration to one where base rocking is allowed, the fundamental period becomes longer. This generally implies a reduction in the spectral acceleration and an increase in the spectral displacement demand (see points RB—i.e., rocking at the base—in Figure 3). At the same time, when the base dynamically rotates, the MR device leads to an increase of dissipated energy, with a further decrease of the acceleration, even accompanied by the reduction of displacement demand (points SA in Figure 3). This the basic concept which the proposed protection strategy is based on. It is similar to the conventional approach of base isolation with additional dissipation, only in this case the technique takes advantage of the rotation of the base, not of the horizontal displacement in the isolators.

The proposed system has two objectives:

(1) Reducing the bending moment demand in the columns;
(2) Limiting the top displacement demand within given limits, to avoid the damage of nonstructural elements as well as to limit detrimental second-order effects.

To this aim, a control algorithm developed by the authors in [22] has been adapted herein and reformulated as in Equation (1), where *M*(*t*) is the base bending moment in the instant of time *t*, *x*(*t*) is the base top displacement, and *ẋ*(*t*) is the top velocity. In the case of precast RC one-storey structures, *M*(*t*), *x*(*t*) and *ẋ*(*t*) are the bending moment at the base of a column, the displacement and velocity at the top of that column, respectively.

**Figure 4:**
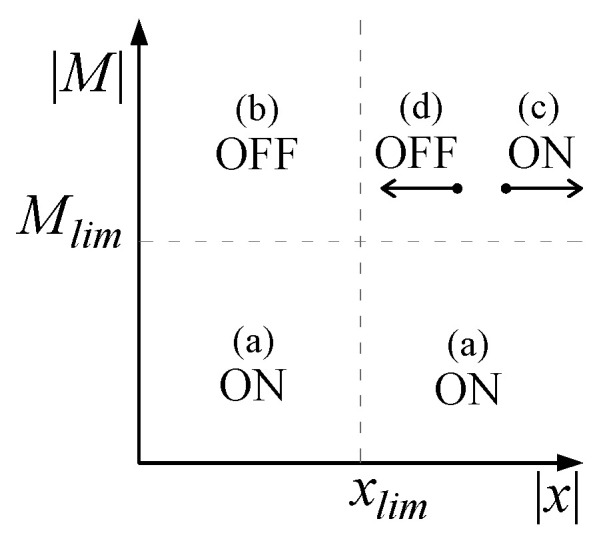
Schematic representation of the control algorithm.

In general, when the base restraint is relaxed (OFF state), the base moment decreases while the top displacement increases, and vice-versa. To keep the base moment and top displacement within predefined limits (*M_lim_* and *x_lim,_* respectively) the control algorithm (Figure 4) switches back and forth from an OFF state (minimum intensity of current *i_min_* to the damper) to an ON state (maximum intensity of current *i_max_* to the damper). It is worth noting that *x_lim_* and *M_lim_* are, more precisely, calibration parameters of the algorithm, whose values, in some instants of time, will be exceeded during the dynamic response of the structure. The structure of the algorithm (Equation (1)) itself contemplates the possibility that said values are exceeded. In such cases, the algorithm “turns off” or “turns on” the device to “run for cover” and to damp down such response peaks. In more detail, when the bending moment is lower than *M_lim_*, the maximum damping is applied (case (a) in Equation (1) and Figure 4) to the system, which then tends to behave like a FB one. When the bending moment is beyond the threshold, three possible scenarios arise:

- For displacements lower than *x_lim_*, the damping is set to the minimum value so as to enable base rocking and to induce energy dissipation (case (b) in Equation (1) and Figure 4);
- When the displacement is greater than *x_lim_* and is still growing (i.e., the velocity *ẋ*(*t*) has the same sign), the damping is set to maximum so as to make the base restraint stiffer and to prevent the displacement from rising again (case (c) in Equation (1) and Figure 4);
- When the displacement is greater than *x_lim_* but is decreasing (i.e., the velocity *ẋ*(*t*) is in the opposite direction), the damping is set to minimum to achieve energy dissipation into the damper (case (d) in Equation (1) and Figure 4).

(1) $$\begin{array}{l} (a)\;\mathrm{if}\;|M\left(t\right)|\;<\;M_{\lim}\;\to \;\mathrm{switch}\;\mathrm{to}\;\mathrm{ON}\\ (b)\;\mathrm{if}\;\left|M\left(t\right)\right|\geq M_{\lim}\;\mathrm{and}\;\;\left|x\left(t\right)\right|<x_{\lim}\;\to \;\mathrm{switch}\;\mathrm{to}\;\mathrm{OFF}\\ (c)\;\mathrm{if}\;|M\left(t\right)|\;\geq \;M_{\lim}\;\mathrm{and}\;\left|x\left(t\right)\right|\;\geq \;x_{\lim}\;\mathrm{and}\;x(t)\;\cdot \;\dot{x}(t)\;>\;0\;\to \;\mathrm{switch}\;\mathrm{to}\;\mathrm{ON}\\ (d)\;\mathrm{if}\;|M\left(t\right)|\;\geq {\;M}_{\lim\;}\mathrm{and}\;|x\left(t\right)|\geq x_{\lim}\;\mathrm{and}\;x(t)\;\cdot \;\dot{x}(t)\;\leq \;0\;\to \;\mathrm{switch}\;\mathrm{to}\;\mathrm{OFF} \end{array}$$

## 3. Calibration of the Control Strategy: Application to a Case Study Structure

The above control strategy needs to be calibrated in order to work effectively. To do that, the following parameters must be set:

- *x_lim_*, limit value displacement at the top of the column;
- *M_lim_*, limit value of base bending moment for the column;
- *k_s_*, stiffness of the base spring;
- *c_ON_*, the damping capability of the MR damper in the ON state (details in Section 3.4);
- *c_OFF_*, the damping capability of the MR damper in the OFF state (details in Section 3.4).

A possible procedure to carry out such a calibration is shown herein (Section 3.4), with reference to a one-story RC precast case study structure. The optimal parameters are those involving, for different ground motions, the maximum reduction of the bending moment at the column base, at the same time ensuring that the top displacement does not exceed that of the FB system.

### 3.1. Case Study Structure

**Figure 5:**
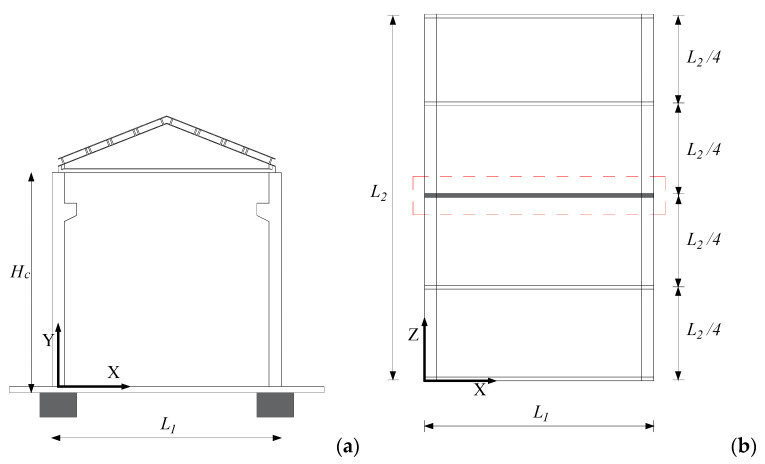
Case study frame structure. The reference 2D frame (**a**) is highlighted in the plan view on the right (**b**).

The case study structure (Figure 5a) is an existing industrial RC precast building designed and built in the 1970′s in Napoli (Italy) without seismic provisions [25]. The plan area of the building is 15 m (L_1_) × 24 m (L_2_), the columns are 9 m high (H_C_), the roof is also prefabricated, with sloping pitches. The building is intended for industrial activities, for this reason a crane with its brackets is placed at 7.5 m from the column base and is considered in the model. The beam has a variable height with a maximum at the middle span, a slope equal to 10%, and a variable width from a T-shape at the supports to a I-shape at the middle. Columns are subjected to the axial force caused by the beam and roof weight. 

The application shown herein as first investigation of the proposed technology is performed for simplicity through 2D non-linear analyses of an intermediate planar frame of the given structure. This frame is illustrated in the plan view in Figure 5. The roof is assumed to behave like a rigid floor. The five transversal frames are all the same, therefore the total seismic mass can be considered equally shared among them.

### 3.2. Seismic Loads

**Figure 6:**
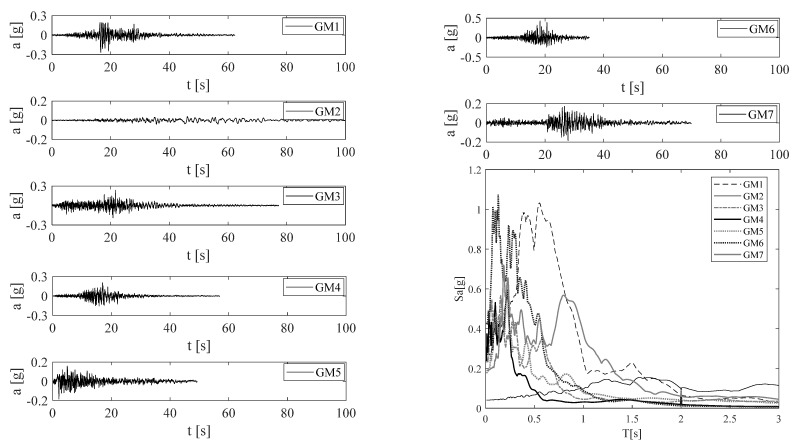
Selected ground motions and spectra.

Nonlinear time-history analyses were performed using seven earthquake ground motions (GMs) (Table 1) selected according to the conditional spectrum approach [26,27] from the NGA West-2 (http://peer.berkeley.edu/ngawest2/ accessed on 15 January 2016) database [28]. The conditioning intensity measure for the record selection was the spectral pseudo-acceleration S_a_(T_1_), derived by the 5% damped spectrum assigned to Napoli (Italy) by the Italian code for a return period T_r_ = 500 years [29]. T_1_ = 2.0 s is the fundamental period of the case study structure in the SA configuration (see next section). Scale factor for each accelerogram is also reported in Table 1. Figure 6 shows the (scaled) acceleration time-history and the elastic spectrum for each of the seven selected GMs.

### 3.3. Numerical Modelling

**Figure 7:**
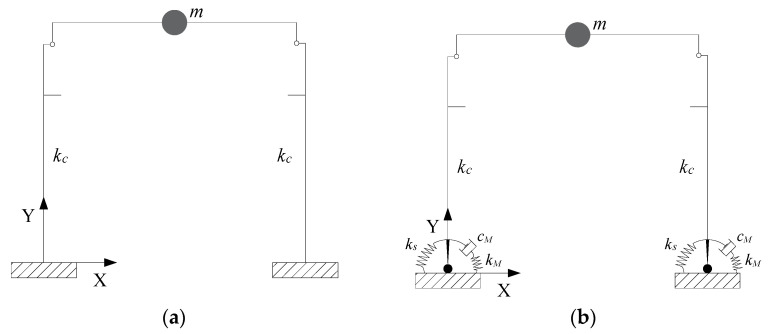
(**a**) Fixed base configuration; (**b**) Damped semi-active control configuration.

The calibration of the control strategy was performed by using two nonlinear models implemented through the software OpenSees (version 2.4.5) [30], one representing the FB structural configuration (Figure 7a), the other representing the SA structural configuration (Figure 7b). 

In the FB configuration, the columns and the main beam were modelled through an elastic element, i.e., the OpenSees [30] elasticBeamColumn element. An equivalent constant cross-section was assigned to the beam, with a height *H_m,beam_* equal to the mean height of the real beam and a width *B_m,beam_* obtained by dividing the mean area *A_mean_* of the beam sections for the mean height *H_m,beam_*. The dimensions of the beam and columns cross-sections were: *B_m,beam_* = 0.25 m, *H_m,beam_* = 1.14 m, *B_column_* = 0.55 m, *H_column_* = 0.55 m. It is worth noting that the software did not allow for modeling the variability of the cross section, however, this did not affect the results since the simplified modeling of the beam ensured the equivalence both in terms of mass and stiffness with the real one. The beam-to-column connection was modelled by a hinge, implemented in OpenSees [30] through an equalDOF constraint assigned to the ends of the elements connected. Since the structural elements were modeled through their longitudinal axis, rigid horizontal and vertical offsets were added to take into account any eccentricities between beam and column axes. The brackets supporting the crane were also modelled as rigid elements. Columns were fixed at the base. The seismic mass accounts for the contribution of beam, columns, tiles, crane-beam and cladding panels. The fundamental period in the FB state is 1.4 s. 

The numerical model in the SA configuration (Figure 7b) was developed as above, with the addition of applying a hinge at the base of columns, together with a rotational spring and a rotational damper installed in parallel. The rotational spring was modelled by an elastic ZeroLengthElement, while the rotational damper was simulated using a ViscousDamper material assigned to another ZeroLengthElement. The latter is represented by a Maxwell model, i.e., a linear spring (stiffness *k_M_*) and a nonlinear dashpot (viscous coefficient *c_M_*) connected in series. The ViscousDamper material was able to simulate the hysteretic response of nonlinear viscous dampers, but also to accurately reproduce the cyclic behavior of MR dampers [24]. The fundamental period of the structure in the SA configuration is 2.0 s.

### 3.4. Calibration of the Controller and Numerical Simulations

The controller was optimized to achieve two goals:

(1) The largest reduction of the bending moment at the base of the columns;
(2) To limit any increase in displacement demand, so that the maximum value was at most equal to that of the fixed base state increased by 30% (allowed tolerance), in any case lower than the yielding displacement (200 mm).

According to that, a trial and error approach was used to find the optimal value for *M_lim_* and *x_lim_* as a function of the peak demand of the FB configuration, *M_max,FB_* and *x_max,FB_* respectively. The result of the optimization procedure is in Equation (2) and it is comparable with that already achieved by the authors for a different application of the same control technique [23]:

(2) $$M_{lim}=0.08\;M_{\max,\mathrm{FB}}\;x_{lim}=0.5\;x_{\max,\mathrm{FB}}$$

where *M_max,FB_* and *x_max,FB_* were assumed to be equal to the mean values of the seven records. The SA base restraint, as known, is made of rotational spring stiffness *k_s_* and a Maxwell element, the latter made of a linear spring *k_MR_* connected in series to a nonlinear viscous damper with a damping coefficient *c_MR_*. The value of the stiffness *k_MR_* in general must be sufficiently large so as to achieve pure damping behavior, however, not too large so as not to cause numerical problems in the integration process. Generally, good results were achieved when the damping coefficient of the damper to non-linear spring stiffness ratio was selected to be one or two degrees less than the time step (Δt) of the analysis. According to this, *k_MR_* was assumed to be that in Equation (3). The viscous component of the Maxwell element with the damping constant *c_MR_* represents the *MR* damper response. According to [24], the reaction force *F_MR_* of such a device can be expressed as in Equation (4), where *ẋ_MR_* the relative velocity between the damper’s ends and *α* = 0.1. However, in the case of a rotational device, it was necessary to rewrite Equation (4) in terms of the bending moment *M_MR_* and rotational velocity $\dot{\theta }\left(t\right)$ as in Equation (5).

(3) $$k_{\mathrm{MR}}=10{\;}_{\mathrm{CMR}}/\Delta t$$

(4) $$F_{\mathrm{MR}}\left(t\right){=-c}_{\mathrm{MR}}\cdot {\left|{\dot{𝒳}}_{\mathrm{MR}}^{}\left(t\right)\right|}^{\alpha }\cdot \mathrm{sign}\left[{\dot{𝒳}}_{\mathrm{MR}}^{}\left(t\right)\right]$$

(5) $$M_{\mathrm{MR}}\left(t\right){=-c}_{\mathrm{MR}}\cdot {\left|{\dot{\theta }}_{\mathrm{MR}}^{}\left(t\right)\right|}^{\alpha }\cdot \mathrm{sign}\left[{\dot{\theta }}_{\mathrm{MR}}^{}\left(t\right)\right]$$

where *c_MR_* is equal to *c_ON_* when the damper is switched ON, equal to *c_OFF_* when it is switched OFF. It is worth noting that in Equation (3) *c_MR_* was set to equal to the maximum value for damping, that is *c_ON_*. According to the previous experience of the authors [23], the extreme values of *c_MR_* were set as follows:

(6) $$c_{\mathrm{ON}}=0.08M_{\max,\mathrm{FB}}\;\mathrm{with}\;M_{\max,\mathrm{FB}}\;\mathrm{in}\;\mathrm{kNm}\;\mathrm{and}\;c_{\mathrm{ON}}\;\mathrm{in}\;\mathrm{kNm}/(\mathrm{rad}/s{)}^{0.1}$$

(7) $$c_{\mathrm{OFF}}=\frac{c_{\mathrm{ON}}}{5000}$$

The last parameter to be calibrated was the stiffness of the rotational spring, *k_s_*. When this stiffness is very low, the system can become too deformable, and displacement demand may rise significantly. In such cases, the spring may not be able to re-centre the column during the excitation, resulting in an uncontrolled increase in base rotation and top displacement in one direction. On the other hand, when the stiffness *k_s_* is too high, the system tends to behave like the FB one, so advantages related to the use of the SA technique are not obtained. According to previous experience, the optimal value should be fixed to be equivalent to the lateral stiffness of the FB column (*k_c_* in Figure 1). The criteria finally adopted to optimize the control system are summarized in Table 2 and Table 3.

**Figure 8:**
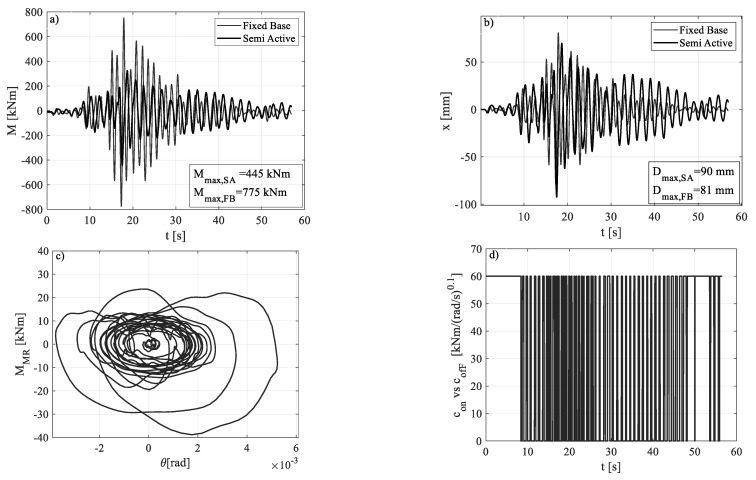
Structural response under GM4: FB vs. SA in terms of bending moment (**a**) and displacement (**b**); MR damper cycles (**c**) and command signal (**d**) for the SA response.

Once the OpenSees non-linear model had been refined with the above values for the involved parameters, the time-history analysis for the SA frame was performed against each of the seven GMs. In the following figures, firstly, the seismic input of medium intensity is shown, that is GM4, whose PGA is nearly the mean value among the seven. In this case, the comparison of the dynamic response of FB and SA cases is shown, together with the cyclic response of MR dampers and the registration of the MR command signal during that excitation (Figure 8). It should be noted that the SA control system leads to a significant reduction in the bending moment demand, with a reduction in peak values from 775 kNm to 445 kNm (43% reduction). This large benefit has a small cost, that is, approximately 10% of the increase in peak displacement demand at the roof. Figure 8c,d shows the activity of the MR device. The number and the width of the moment-rotation cycles in the damper illustrate the amount of energy dissipated into the device. Finally, from the command signal time-history (Figure 8d) one can observe that in the first phase of the seismic input the MR damper is normally switched ON to reproduce a base restraint like the FB case. When the significant part of the ground motion starts (at approximately 10 s), the series of ON and OFF commands begins, according to the logic of the Equation (1).

The percent variations of maximum bending moment and top displacement in the SA configuration, with respect to the FB configuration, against all the seven GMs, are listed in Table 4. With regard to the peak base bending moment, the SA control technique led to a percentage reduction in the range of 19–73%, with a mean of 45%. With regard to the top displacement demand, on the other hand, a decrease was registered against some of the earthquakes (#1, #3, #7). This was due to the amount of energy dissipated into the MR dampers and results in the range of 13–47%. Other seismic inputs (#2, #4, #5, #6) caused an increase in displacement demand, however it was approximately at the set allowable value of 30%. This confirms that the control logic was designed properly and that it achieved the desired goals.

**Figure 9:**
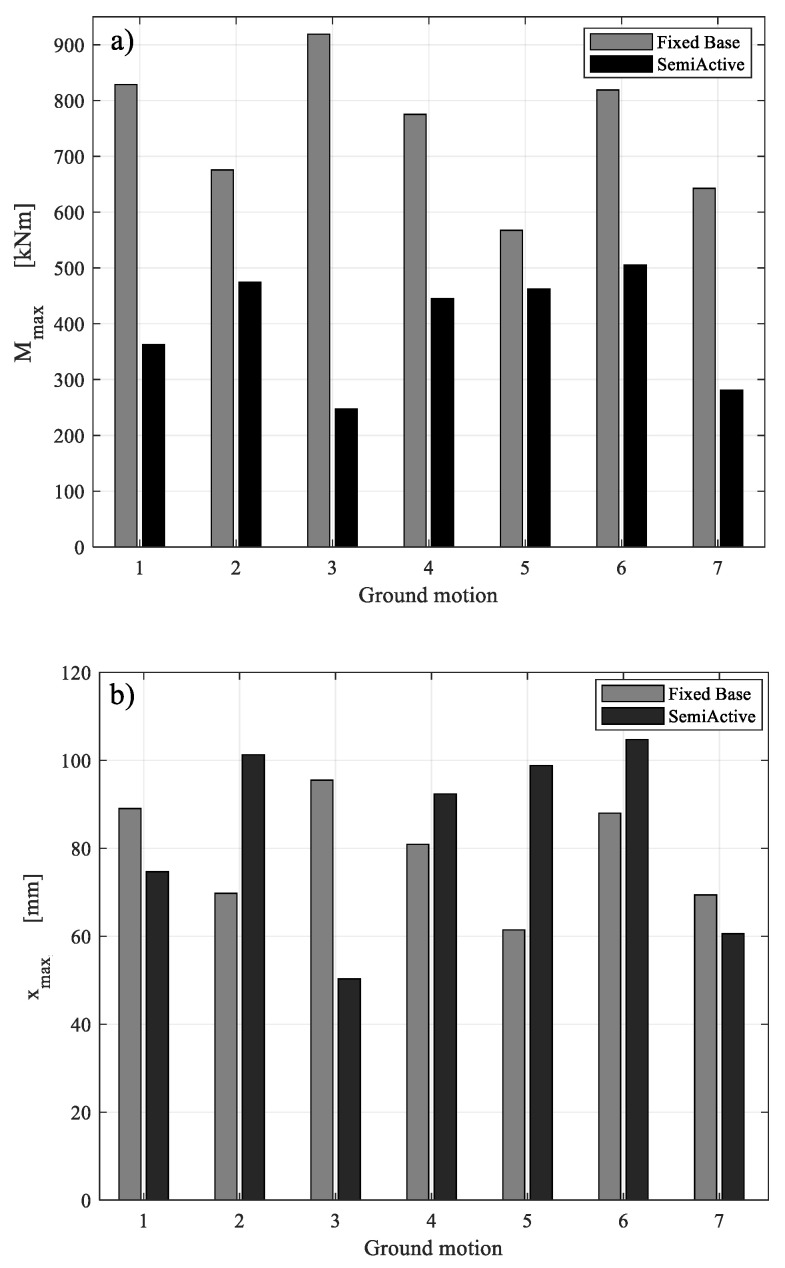
FB versus SA in terms of maximum bending moment (**a**) and of maximum roof displacement (**b**).

**Figure 10:**
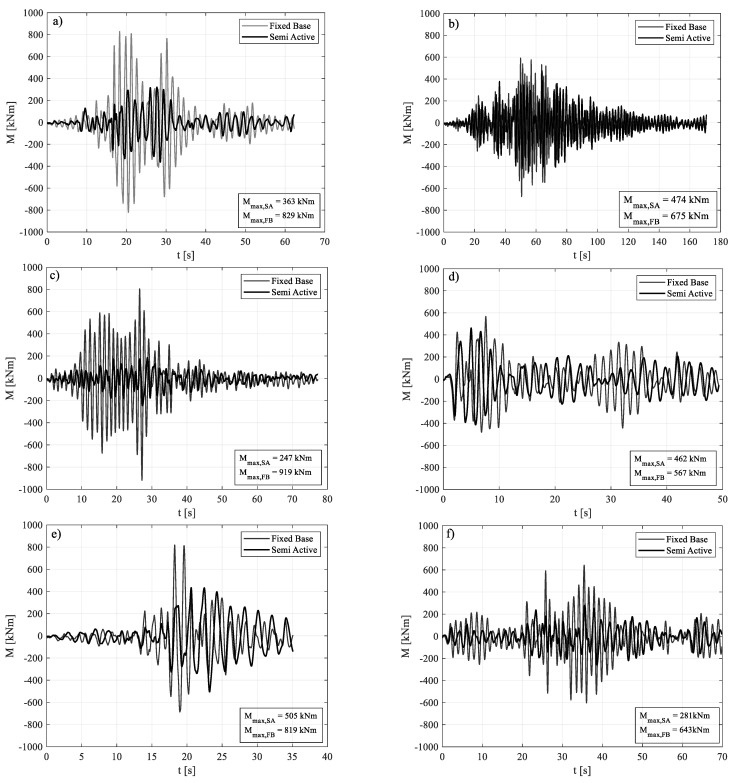
FB vs. SA, in terms of bending moment: (**a**) GM 1; (**b**) GM 2; (**c**) GM 3; (**d**) GM 5; (**e**) GM 6; (**f**) GM 7.

**Figure 11:**
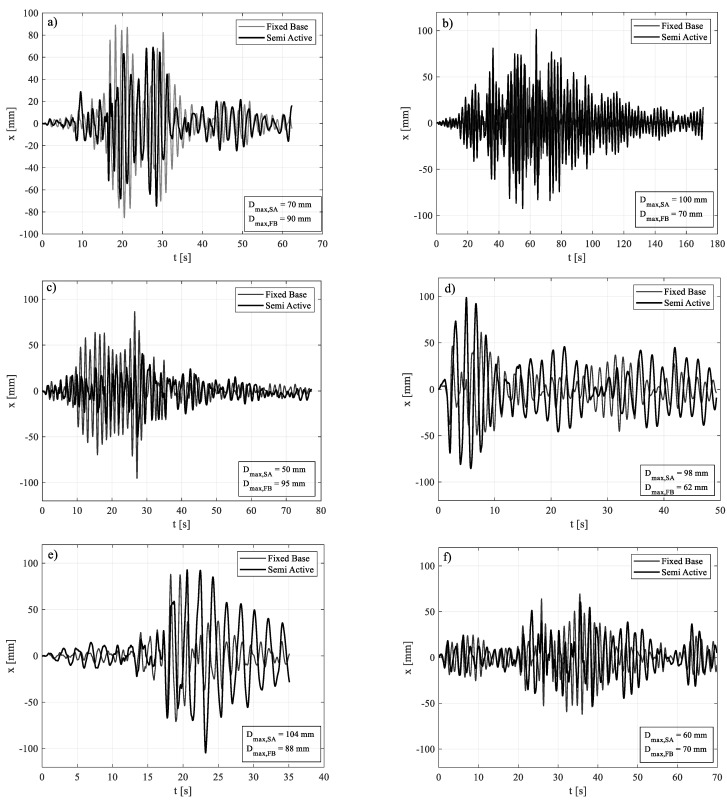
FB vs. SA, in terms of top displacement: (**a**) GM 1; (**b**) GM 2; (**c**) GM 3; (**d**) GM 5; (**e**) GM 6; (**f**) GM 7.

The comparison between the maximum value of the base bending moment and top displacement in FB and SA configurations, for each ground motion, is shown in Figure 9. Figure 10 and Figure 11, instead, show the time histories of the bending moment and the top displacement, for the seven seismic inputs, except for GM4 already shown in Figure 8.

## 4. Conclusions

An innovative semi-active strategy for single-story RC precast buildings has been presented. It aimed at significantly reducing the base bending moment due to seismic actions, as well as controlling lateral displacement, to reduce induced damage even to non-structural components. A specific control algorithm was adopted to drive MR dampers installed at the base of the columns, in parallel with elastic springs, to achieve the above goals. It is based on a physically-sound approach, and is easy to use and able to be implemented in real case applications. An optimization procedure of the involved parameters was also drawn and tested through a case-study application.

The case-study structure was a one-story industrial RC precast building. The SA response against seven earthquakes achieved the structural goals the control logic is designed for. In particular, the bending moment reduction on average was 45% compared to the fixed base configuration. In three of the cases, this was also accompanied by a displacement demand reduction due to a large amount of SA dissipated energy, while for the remaining seismic inputs an increase was registered, however at approximately the allowable value (30%). Finally, it is worth noting that the maximum displacement result was lower than the RC column yielding displacement as well as lower than the displacement threshold value corresponding to the damage limit state according to the Italian code [29].

The above analyses were carried out with a hypothesis of rigid in-plane behavior of the deck and by carrying out 2D response analyses in the plane of the main frames. This preliminary study requires further investigation for several different assumptions. Additionally, further investigation regarding implementation and the possible use of 3D applications is required, as well as consideration of issues related to soil type, seismic input directivity and vertical seismic accelerations [31,32]. The authors are working to produce new developments, however, the results achieved in the numerical simulations so far highlight the very strong potential of the proposed technology.

## Figures and Tables

**Table 1:** Main characteristics of seismic inputs for non-linear analyses.

No.	Record Serial Number	Earthquake	Year	Station Name	Magnitude M_w_	Epicentral Distance [km]	PGA [g]	SF [-]
1	1304	Chi-Chi, Taiwan	1999	HWA059	7.62	70	0.132	1.9
2	1398	Chi-Chi, Taiwan	1999	KAU087	7.62	147	0.030	1.3
3	1788	Hector Mine	1999	Hemet Fire Station	7.13	117	0.058	3.6
4	1783	Hector Mine	1999	Fort Irwin	7.13	84	0.116	1.6
5	15	Kern County	1952	Taft Lincoln School	7.36	43	0.164	1.0
6	1067	Northridge-01	1994	San Bernardino-CSUSB Gr	6.69	114	0.052	6.2
7	3823	Hector Mine	1999	Universal City-Hwy 101	7.13	200	0.035	5.2

**Table 2:** Optimal calibration criteria for the parameters involved in the proposed SA control technique.

Parameter	Criterion for Optimal Value *
*M_lim_*	0.08 *M_max,FB_*
*x_lim_*	0.5 *x_max,FB_*
*c_ON_*	0.08 *M_max,FB_*
*c_OFF_*	*c_ON_/*5000
*k_M_*	10 *c_ON_/*Δ*t*
*k_s_*	*k_c_*

* Δt, sampling time for the numerical analyses; *k_c_*, lateral stiffness of the RC column.

**Table 3:** Optimal calibration of the SA control technique for the case-study application.

GM	*M_max,FB_*	*x_max,FB_*	Control System Optimal Parameters	
	[kNm]	[mm]		
1	829	90	*M_lim_*	60 kNm
2	675	70	*x_lim_*	40 mm
3	919	95	*c_ON_*	60 kNm/(rad/s)^0.1^
4	775	81	*c_OFF_*	0.012 kNm/(rad/s)^0.1^
5	567	62	*k_M_*	1.2 MNm/rad
6	819	88	*k_c_*	86 MNm/rad
7	643	70	*k_s_*	86 MNm/rad
mean	747	79		

**Table 4:** Peak values of the response for each GM and percentage variation of the SA vs. FB configuration (minus means reduction).

GM	*M_max,FB_*	*M_max,SA_*	*%*	*x_max,FB_*	*x_max,SA_*	*%*
-	[kNm]	[kNm]	-	[mm]	[mm]	-
1	829	363	−56	90	70	−16
2	675	474	−30	70	100	31
3	919	247	−73	95	50	−47
4	775	445	−43	81	90	11
5	567	462	−19	62	98	32
6	819	505	−38	88	104	16
7	643	281	−56	70	60	−13

## Data Availability

The data presented in this study are available on request from the corresponding author.

## References

[1] Magliulo G., Ercolino M., Petrone C., Coppola O., Manfredi G. (2014). Emilia Earthquake: The Seismic Performance of Precast RC Buildings. Earthq. Spectra.

[2] Ercolino M., Magliulo G., Manfredi G. (2016). Failure of a precast RC building due to Emilia-Romagna earthquakes. Eng. Struct..

[3] Keshtegar B., Nehdi M.L., Trung N.-T., Kolahchi R. (2021). Predicting load capacity of shear walls using SVR–RSM model. Appl. Soft Comput..

[4] Marriott D., Pampanin S., Bull D., Palermo A. (2007). Improving the seismic performance of existing reinforced concrete buildings using advanced rocking wall solutions. Proceedings of the 2007 NZSEE Conference.

[5] Mazza F. (2021). Dissipative steel exoskeletons for the seismic control of reinforced concrete framed buildings. Struct. Control Health Monit..

[6] Al-Furjan M., Hajmohammad M.H., Shen X., Rajak D.K., Kolahchi R. (2021). Evaluation of tensile strength and elastic modulus of 7075-T6 aluminum alloy by adding SiC reinforcing particles using vortex casting method. J. Alloy. Compd..

[7] Casotto C., Silva V., Crowley H., Nascimbene R., Pinho R. (2015). Seismic fragility of Italian RC precast industrial structures. Eng. Struct..

[8] Magliulo G., Cimmino M., Ercolino M., Manfredi G. (2017). Cyclic shear tests on RC precast beam-to-column connections retrofitted with a three-hinged steel device. Bull. Earthq. Eng..

[9] Martinelli P., Mulas M.G. (2010). An innovative passive control technique for industrial precast frames. Eng. Struct..

[10] Minghini F., Tullini N. (2021). Seismic retrofitting solutions for precast RC industrial buildings struck by the 2012 earth-quakes in Northern Italy. Front. Built Environ..

[11] Murahidy A.G., Spieth H.A., Carr A.J., Mander J.B. (2004). Design, construction and dynamic testing of a post-tensioned pre-cast reinforced concrete frame building with rocking beam-column connections and ADAS Elements. Proceedings of the 2004 NZSEE Conference.

[B12-materials-15-00759] 12.Decreto legge 6 giugno 2012 n. 74, Interventi urgenti in favore delle popolazioni colpite dagli eventi sismici che hanno interessato il territorio delle province di Bologna, Modena, Ferrara, Mantova, Reggio Emilia e Rovigo, il 20 e il 29 maggio 2012. (In Italian)

[13] Pampanin S. (2012). Reality-check and renewed challenges in earthquake engineering. Bull. N. Z. Soc. Earthq. Eng..

[14] Spieth H., Arnold D., Davies M., Mander J., Carr A. (2004). Seismic performance of post-tensioned precast concrete beam to column connections with supplementary energy dissipation. Proceedings of the NZSEE Conference.

[15] Belleri A., Schoettler M.J., Restrepo J.I., Fleishman R.B. (2014). Dynamic Behavior of Rocking and Hybrid Cantilever Walls in a Precast Concrete Building.

[16] Lu L., Liu X., Chen J., Lu X. (2016). Seismic performance of a controlled rocking reinforced concrete frame. Adv. Struct. Eng..

[17] Masrom M.A., Hamid N.H.A. (2020). Review on the rocking wall systems as a self-centering mechanism and its interaction with floor diaphragm in precast concrete structures. Lat. Am. J. Solids Struct..

[18] Zhao Z., Su X. (2021). Seismic Response Analysis of Prestressed Concrete Rocking Frame. Appl. Sci..

[19] Marriott D., Pampanin S., Bull D., Palermo A. (2008). Dynamic testing of precast, post-tensioned rocking wall systems with alternative dissipating solutions. Bull. New Zealand Soc. Earthq. Eng..

[20] Jiang Q., Wang H., Feng Y., Chong X., Huang J., Liu Y. (2020). Enhancing the Seismic Performance of Precast RC Frames with Cladding Panels through Setting U-Shaped Dampers and Rocking Walls. Shock. Vib..

[21] Kurama Y.C., Sritharan S., Fleischman R.B., Restrepo J.I., Henry R.S., Cleland N.M., Ghosh S.K., Bonelli P. (2018). Seismic-Resistant Precast Concrete Structures: State of the Art. J. Struct. Eng..

[22] Caterino N. (2015). Semi-active control of a wind turbine via magnetorheological dampers. J. Sound Vib..

[23] Caterino N., Georgakis C.T., Spizzuoco M., Occhiuzzi A. (2016). Design and calibration of a semi-active control logic to mitigate structural vibrations in wind turbines. Smart Struct. Syst..

[24] Caterino N., Spizzuoco M., Occhiuzzi A. (2011). Understanding and modelling the physical behaviour of magnetorheologi-cal dampers for seismic structural control. Smart Mater. Struct..

[25] Magliulo G., Fabbrocino G., Manfredi G. (2008). Seismic assessment of existing precast industrial buildings using static and dynamic nonlinear analyses. Eng. Struct..

[26] Lin T., Haselton C.B., Baker J.W. (2013). Conditional spectrum-based ground motion selection. Part I: Hazard consistency for risk-based assessments. Earthq. Eng. Struct. Dyn..

[27] Lin T., Haselton C.B., Baker J.W. (2013). Conditional spectrum-based ground motion selection. Part II: Intensity-based assessments and evaluation of alternative target spectra. Earthq. Eng. Struct. Dyn..

[28] Iervolino I., Spillatura A., Bazzurro P. RINTC project: Assessing the (implicit) seismic risk of code-conforming structures in Italy. Proceedings of the ECCOMAS Thematic Conference on Computational Methods in Structural Dynamics and Earthquake Engineering.

[29] Ministero delle Infrastrutture (2018). D.M. 17/01/2018—Norme Tecniche per le Costruzioni, G.U. n. 42/2018.

[30] McKenna F., Fenves G.L., Scott M.H. (2000). OpenSees: Open System for Earthquake Engineering Simulation.

[31] Chen Z., Yang P., Liu H., Zhang W., Wu C. (2019). Characteristics analysis of granular landslide using shaking table model test. Soil Dyn. Earthq. Eng..

[32] Zhang W.G., Meng F.S., Chen F.Y., Liu H.L. (2021). Effects of spatial variability of weak layer and seismic random-ness on rock slope stability and reliability analysis. Soil Dyn. Earthq. Eng..

